# Strong morphological defects in conditional *Arabidopsis abp1* knock-down mutants generated in absence of functional ABP1 protein

**DOI:** 10.12688/f1000research.7654.1

**Published:** 2016-01-20

**Authors:** Jaroslav Michalko, Matouš Glanc, Catherine Perrot-Rechenmann, Jiří Friml

**Affiliations:** 1 Institute of Science and Technology Austria, Klosterneuberg, Austria; 2 Institute of Plant Genetics and Biotechnology, Slovak Academy of Sciences, Nitra, Slovakia; 3 Department of Experimental Plant Biology, Faculty of Sciences, Charles University, Prague, Czech Republic; 4 Institut Jean-Pierre Bourgin, INRA, AgroParisTech, CNRS, Université Paris-Saclay , Versailles, France

**Keywords:** Arabidopsis, auxin, AUXIN BINDING PROTEIN 1 (ABP1), knock-down mutant, off-target

## Abstract

The Auxin Binding Protein 1 (ABP1) is one of the most studied proteins in plants. Since decades ago, it has been the prime receptor candidate for the plant hormone auxin with a plethora of described functions in auxin signaling and development. The developmental importance of ABP1 has recently been questioned by identification of *Arabidopsis thaliana abp1* knock-out alleles that show no obvious phenotypes under normal growth conditions. In this study, we examined the contradiction between the normal growth and development of the *abp1* knock-outs and the strong morphological defects observed in three different ethanol-inducible *abp1* knock-down mutants ( *abp1-AS, SS12K, SS12S*). By analyzing segregating populations of *abp1* knock-out vs. *abp1* knock-down crosses we show that the strong morphological defects that were believed to be the result of conditional down-regulation of ABP1 can be reproduced also in the absence of the functional ABP1 protein. This data suggests that the phenotypes in *abp1* knock-down lines are due to the off-target effects and asks for further reflections on the biological function of ABP1 or alternative explanations for the missing phenotypic defects in the *abp1* loss-of-function alleles.

## Introduction

The naturally occurring auxin, indole-3-acetic acid, plays a central role in plant growth and development alone or in orchestration with other plant hormones. Proper sensing and interpretation of fluctuating cellular auxin signals is necessary for mediating a diverse range of developmental and cell biology responses ( Enders & Strader, 2015; Grunewald & Friml, 2010; Paciorek *et al.*, 2005; Petrasek *et al.*, 2006). In the early screens for auxin receptors, Auxin Binding Protein 1 (ABP1) has been identified based on its ability to bind auxin with high affinity ( Hertel *et al.*, 1972; Löbler & Klämbt, 1985) and soon became a prime candidate for an extracellular auxin receptor based mainly on electrophysiological studies utilizing antibodies against ABP1 that showed rapid ABP1-mediated modulation of plasma membrane ion transport in an early step of auxin action ( Barbier-Brygoo *et al.*, 1989; Leblanc *et al.*, 1999). Over the next decades, the auxin-binding activity of ABP1 has been characterized in detail by biochemical studies ( Batt *et al.*, 1976; Napier *et al.*, 2002; Napier & Venis, 1995; Ray *et al.*, 1977) and its protein structure including the auxin-binding pocket has been revealed ( Woo *et al.*, 2002). Phylogenetic studies have shown that ABP1 homologues are present in the genomes of all plant species from bryophytes to flowering plants ( Tromas *et al.*, 2010) with more than one copy present e.g. in the genome of maize, rice, poplar or the moss *Physcomitrella patens* ( http://phytozome.jgi.doe.gov/pz/portal.html).

Since its discovery, however, the biological importance of the ABP1 protein as a plasma membrane auxin receptor has been a matter of debates, in part because of its predominant subcellular localization in the endoplasmic reticulum (ER) in maize where the conditions for auxin binding are unfavorable ( Habets & Offringa, 2015; Napier *et al.*, 2002). Recently, these discussions were revived by the isolation of two new *Arabidopsis abp1* knock-out alleles, *abp1-c1* and *abp1-TD1* ( Gao *et al.*, 2015) that show no obvious phenotypes under standard growth conditions. The contradiction between this observation and the previously published embryo-lethal phenotypes of *abp1* mutants ( Chen *et al.*, 2001; Tzafrir *et al.*, 2004) has recently been clarified by proving that the embryo-lethality of the originally reported alleles *abp1-1* and *abp1-1s* was caused by disruption of the tightly-linked neighboring gene *BELAYA SMERT (BSM)* rather than knock-out of *ABP1* ( Dai *et al.*, 2015; Michalko *et al.*, 2015). This correction and the demonstration of normal embryo development in the *abp1* knock-outs ( Michalko *et al.*, 2015) suggest that ABP1 plays no essential role in early *Arabidopsis* embryogenesis.

The ongoing discussion focuses on the relevance of *ABP1* in auxin signaling and other post-embryonic auxin-related biological processes that have been demonstrated using different genetic tools, namely the conditional knock-down (KD) lines, the *abp1-5* weak allele harboring a point mutation in the ABP1 auxin-binding pocket and gain-of-function alleles, all of which often provided internally consistent results ( Braun *et al.*, 2008; Čovanová *et al.*, 2013; David *et al.*, 2007; Grones *et al.*, 2015; Robert *et al.*, 2010; Sassi *et al.*, 2014; Tromas *et al.*, 2013; Xu *et al.*, 2010; Xu *et al.*, 2014).

Conditional *ABP1* KD lines *SS12S6*, *SS12K9* and *abp1-AS* have been generated using two fundamentally different approaches of gene or protein down-regulation. In the *SS12S6* and *SS12K9* lines, ABP1 was inactivated by inducible over-expression of a recombinant immunoglobulin fragment termed single-chain fragment variable (scFv) ( Conrad & Fiedler, 1998). This construct, consisting of the heavy- and light-chain variable domains of a well-characterized anti-ABP1 monoclonal antibody mAb12 ( David & Perrot-Rechenmann, 2001; David *et al.*, 2007; Leblanc *et al.*, 1999) linked by a flexible peptide was additionally fused to the sequence encoding the 3‘KDEL motif to mediate scFv ER-retention in the *SS12K9* line, while the *SS12S*-encoded scFv12 was meant to be secreted to the apoplast. *In planta-*produced scFv12 was able to pull down ABP1, and reciprocally immuno-precipitation of ABP1 using another antibody was shown to pull down scFv12 ( Tromas *et al.*, 2009). An antisense approach was utilized in the *abp1-AS* line, where inducible over-expression of full-length *ABP1* antisense cDNA led to the formation of duplexes with its sense mRNA, thus preventing ABP1 translation, and potentially also transcription by RNA interference mechanism ( Meister & Tuschl, 2004; Tufarelli *et al.*, 2003). Both antibody- and antisense-based lines use the ethanol-inducible system, which is well established and widely used for the conditional expression of plant genes ( Deveaux *et al.*, 2003; Roslan *et al.*, 2001).

These three *abp1* knock-down lines have been instrumental to connect ABP1 function to multiple cellular and developmental processes. For example, they showed defects in shoot and root growth ( Braun *et al.*, 2008; Tromas *et al.*, 2009), cell wall re-modeling ( Paque *et al.*, 2014) or clathrin-mediated endocytosis of PIN auxin efflux carriers ( Dhonukshe *et al.*, 2007; Robert *et al.*, 2010). In contrast, the *abp1* gain-of-function transformants promote PIN internalization both in tobacco and *Arabidopsis* ( Grones *et al.*, 2015; Robert *et al.*, 2010). Contrasting effects of *ABP1* KD and gain-of-function lines were shown also in the case of auxin effect on the control of leaf epidermal pavement cells morphogenesis ( Braun *et al.*, 2008; Nagawa *et al.*, 2012) on ROP GTPase activation ( Xu *et al.*, 2010) and on microtubule rearrangement ( Chen *et al.*, 2014; Xu *et al.*, 2014). Furthermore, analysis of ABP1 variants with mutations in the auxin-binding pocket demonstrated the importance of auxin-binding to ABP1 for its gain-of-function phenotypes ( Grones *et al.*, 2015). Altogether, these studies provided an internally consistent picture showing involvement of ABP1 signaling in multiple physiological and cellular processes. These observations were further supported by the finding that loss-of-function mutants in *TMK* receptor-like protein kinases, that were recently shown to interact with ABP1 in an auxin-inducible manner, show similar phenotypes with *abp1* KD mutants ( Xu *et al.*, 2014) which was consistent with the importance of the ABP1/TMK complex-mediated auxin perception in plant development. Recent identification of wild-type looking *Arabidopsis abp1* loss-of-function alleles by Gao *et al.* (2015) thus questions the interpretation of data obtained in the aforementioned studies.

Here, we address the missing phenotypes in the true *abp1* null alleles in relation to the strong and consistent morphological defects observed in the conditional *abp1* knock-down lines. We show that the morphological phenotypes in *SS12S6, SS12K9* and *abp1-AS* can be generated in the absence of functional ABP1 protein and we discuss possible underlying causes of this.

## Material and methods

### Plant material and growth conditions

*Arabidopsis thaliana* mutants used in this study were: *abp1-c1, abp1-TD1* ( Gao *et al.*, 2015), *abp1-AS, SS12K9, SS12S6* ( Braun *et al.*, 2008; David *et al.*, 2007). *A. thaliana* Col-0 wild type seeds were obtained from The Nottingham *Arabidopsis* Stock Centre (NASC, http://www.arabidopsis.info). For *in vitro* experiments, seeds were surface-sterilized with chlorine vapor, vernalized for 2 days in the dark at 4°C and grown on 1/2 MS 0.8% agar medium with or without 1% w/v sucrose (pH 5.9) on vertical Petri dishes under long day conditions (16 h light/8 h dark) or in complete darkness at 21°C. A sterilized microtube with 500 µl 5% ethanol was placed at the bottom of the plate to induce expression of *abp1-AS, SS12K9* and *SS12S6* constructs before germination. Plates with 5-day old etiolated or 7-day old light-grown seedlings were scanned on a flatbed scanner, phenotyped by visual examination and used for DNA/RNA extraction.

### Genotyping mutants

Ethanol-inducible ABP1 down-regulating lines ( *abp1-AS, SS12K9, SS12S6*) were genotyped for the presence of the *alcR* gene encoding the transcriptional regulator of the ethanol-inducible system using primers *alcR_for* and *alcR_rev* ( Table 1). Fragments amplified from *abp1-c1* with primer pairs ABP1-U409F + ABP1-586R or ABP1-5P + ABP1-586R were digested with *BslI,* which cuts the WT fragment once and does not cut the mutant fragment; *abp1-TD1* was genotyped as described previously ( Gao *et al.*, 2015). Genomic DNA was isolated using the CTAB extraction method. GoTaq G2 polymerase (Promega) and Bio-Rad T100 Thermal Cycler were used for PCR under following conditions: initial denaturation 5 min 98°C; 35–45 cycles (denaturation 30 s at 98°C; annealing 30 s at 55°C, elongation 1 min at 72°C); final elongation 5 min at 72°C. Restriction analysis was performed by adding the restriction enzyme directly to unpurified PCR reaction. Alternatively, Phire Plant Direct PCR Kit (Thermo Scientific by Finnzymes) and QIAquick Gel Extraction Kit (QIAGEN) were used following manufacturer’s instructions to genotype the *SS12K9 x abp1-c1* line.

**Table 1.** Primer sequences used in this study.

ABP1-U409F	CCTCATCACACAACAAAGTCACTC
ABP1-586R	GGAGCCAGCAACAGTCATGTG
ABP1-5P	ATGATCGTACTTTCTGTTGGTTCC
ABP1-2E	TTGCCAATCGTGAGGAATATTAG
pSKTAIL-L3	ATACGACGGATCGTAATTTGTCG
AlcR F	AGAACAAAGAAAGCCAGGA
AlcR R	GCGTGAGAGAAAAGATGA
TUB2 F	TAACAACTGGGCCAAGGGACAC
TUB2 R	ACAAACCTGGAACCCTTGGAGAC

### Quantitative RT-PCR

Total RNA from approximately twenty 8-day old seedlings frozen in liquid nitrogen was extracted using the TRIzol reagent (Invitrogen, Carlsbad, CA, USA) and purified using RNeasy Mini Kit (Qiagen) according to manufacturer’s instructions. 2 µg of purified total RNA were used for a reverse transcription reaction using the iScript cDNA Synthesis Kit (BioRad). qRT-PCR was performed using the LightCycler 480 SYBR Green I Master chemistry (Roche) in a LightCycler480 II thermal cycler (Ser. no. 5659, Roche) according to manufacturer’s instructions. cDNA diluted 1:10 in water was used as a template to prepare 5 µL reaction mixture (final volume). Primers used for the quantitative RT-PCR were designed using QuantPrime ( http://www.quantprime.de). The *ABP1* cDNA fragment (84 bp in length) was amplified with ABP1-2E and ABP1-586R primers. *Arabidopsis Tubulin beta chain 2* ( *TUB2*, At5g62690) amplified with TUB2-F and TUB2-R primers was used as a reference gene ( Dataset 1). Gene expression was calculated with the 2 ^-ΔΔCT^ method ( Livak & Schmittgen, 2001). Results are expressed as the average +/- standard deviation of 2 biological and three technical replicates. Sequences of primers used for genotyping and qRT-PCR analysis are listed in Table 1.

## Results

**Figure 1.**
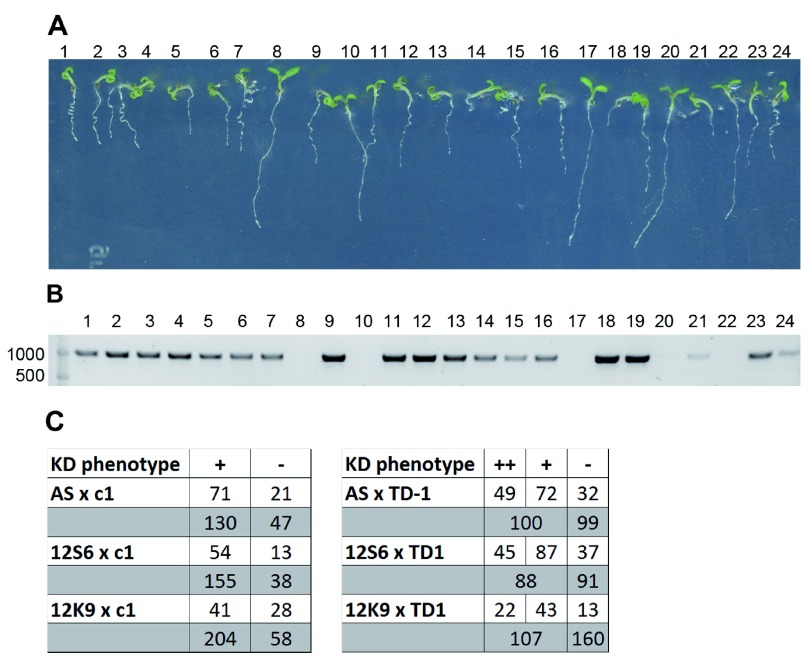
Strong morphological defects in conditional *abp1* knock-down lines correlate with the presence of the ethanol-inducible cassette and segregate normally when crossed with *abp1-c1* knock-out allele. ( **A**) *abp1-AS* × *abp1-c1* F2 plants grown for 7 days in the presence of 5% ethanol segregate strong morphological defects characteristic of the *abp1* conditional knock-down (KD) alleles approximately in a 3:1 ratio. ( **B**) *alcR*-specific PCR bands amplified from the genomic DNA of *abp1-AS* × *abp1-c1* F2 plants shown in ( **A**) demonstrate that the KD phenotype is caused by the presence of the ethanol-inducible insertion. ( **C**) Phenotypes of the scFv12-based KD lines segregate similarly in F2 crosses with *abp1-c1,* while altered segregation ratios can be observed in F2 of all three KD alleles crossed to *abp1-TD1*, which is most apparent in seedlings grown for 5 days in the dark (grey background).

**Figure 2.**
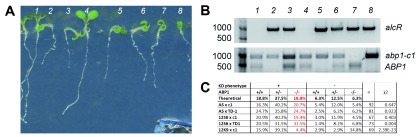
Mendelian segregation of strong ethanol-inducible phenotypes in the F2 generation of *abp1* knock-out × knock-down crosses is independent of *abp1* mutant background. ( **A**) Representative *abp1-AS* × *abp1-c1* F2 plants, ( **B**) PCR products amplified from their genomic DNA and ( **C**) segregation ratios from all crosses show that the ethanol-inducible phenotypes segregate independently of the presence of *abp1* knock-out alleles following approximately Mendelian rules for di-hybrid crosses. Homozygous *abp1* knock-out mutants with the inducible KD phenotype could be found in all crosses (plants 2,5,8 in ( **A**) and ( **B**), red numbers in ( **C**)), suggesting that the phenotype does not require a functional *ABP1* gene. Strong deviations from the expected Mendelian segregation were detected in the *SS12K9* × *abp1-c1* cross, indicating genetic linkage between *ABP1* locus and the inserted ethanol-inducible scFv construct.

Scans of ethanol-induced F2 seedlings of crosses (A) *SS12S6 × abp1-c1*, (B) *SS12S6 × abp1-TD1*, (C) *abp1-AS × abp1-c1*, (D) *abp1-AS × abp1-TD1*, (E) *SS12K9 × abp1-c1* and (F) *SS12K9×abp1-TD1* that were used for phenotyping and genotyping (Figure 1 and Figure 2) Click here for additional data file. Copyright: © 2016 Michalko J et al. 2016 Data associated with the article are available under the terms of the Creative Commons Zero "No rights reserved" data waiver (CC0 1.0 Public domain dedication).

**Figure 3.**
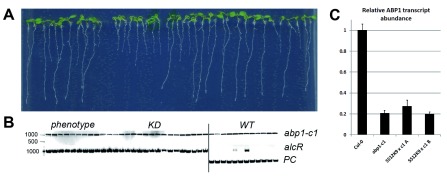
Strong inducible knock-down phenotypes in the absence of functional *ABP1* gene confirmed in the *SS12K9 × abp1-c1* F3 progeny. ( **A**) Representative seedlings of the ethanol-induced F3 progeny of one of the *SS12K9* × *abp1-c1* F2 plants (plant A) that showed KD phenotype in the absence of the functional *ABP1*. All F3 seedlings manifesting KD phenotype were homozygous for *abp1-c1* mutation. ( **B**) Genotyping of the plants shown in **A**. The image is assemled from different regions of two gels that were copy-pasted next to each other in order to save space. ( **C**) qRT-PCR analysis of KD-phenotype positive F3 seedlings of both lines revealed that *ABP1* transcript levels are reduced by about 80% like in the original *abp1-c1* mutant. Altogether these data confirm that in the *abp1* down-regulating lines the KD phenotype can be manifested without the ABP1 function. In ( **C**) average of two biological and three technical replicates +/- SD is shown. PC- positive control.

Agarose gel images from the PCR genotyping of the F2 crosses (A) *SS12S6 × abp1-c1*, (B) *SS12S6 × abp1-TD1*, (C) *abp1-AS × abp1-c1*, (D) *abp1-AS × abp1-TD1*, (E) *SS12K9 × abp1-c1* and (F) *SS12K9 ×abp1-TD1* (Figure 3) All crosses were genotyped for the presence of the *alcR* transcriptional regulator (first row of the gel images) which is an integral part of the ethanol-inducible cassette in *abp1* knock-down lines. The presence of point mutation in *abp1-c1* crosses was genotyped by restriction analysis of *ABP1* PCR product as described in Gao *et al*. (2015) (second row of the gel images of *abp1-c1* crosses). The presence of the T-DNA insertion in *abp1-TD1* crosses was genotyped according to Gao *et al.* (2015) (second and third row of gel images of *abp1-TD1* crosses). GeneRuler DNA ladder mix #0331 (Thermo Scientific) was used as a fragment size standard to determine the approximate size of DNA fragments. Fragment sizes of 1000 bp and 500 bp are indicated. Click here for additional data file. Copyright: © 2016 Michalko J et al. 2016 Data associated with the article are available under the terms of the Creative Commons Zero "No rights reserved" data waiver (CC0 1.0 Public domain dedication).

Source qPCR data (Figure 3c) Individual samples are annotated with their position on a 384-well plate (column A), the cDNA (column B) and primer pair (column C); the Cp value of each sample is shown in column D. The experiment was performed in two biological (1 or 2 at the last position in column B) and three technical replicates. Figure 3c shows gene expression calculated with the 2 ^-ΔΔCT^ method ( Livak & Schmittgen, 2001) from values of ABP1-2E and TUB as a reference gene; using ABP1-5P and/or EF as a reference gene instead gave similar results. Click here for additional data file. Copyright: © 2016 Michalko J et al. 2016 Data associated with the article are available under the terms of the Creative Commons Zero "No rights reserved" data waiver (CC0 1.0 Public domain dedication).

### Segregation of strong morphological defects in conditional *abp1* knock-down alleles crossed with *abp1-TD1* and *abp1-c1* knock-out alleles

To investigate the contradiction between missing phenotypic defects in the loss-of-function *abp1* alleles and strong morphological defects of conditional *ABP1* down-regulating lines (knock-down; KD), we decided to cross both types of lines to test three possible scenarios: 1) The absence of the strong morphological defects in the *abp1-c1* or *abp1-TD1* alleles is caused by an adaptation of the plants to the permanent loss of the ABP1 function, which compensates for this deletion; 2) the strong morphological phenotypes induced in the KD lines do not require functional ABP1 and are caused by off-target effects; or 3) both *abp1-TD-1* and *abp1-c1* lines contain background mutation(s) that suppress the phenotypes caused by the absence of ABP1.

**Supplementary Figure 1.**
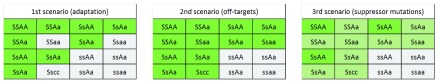
Theoretical genotype and phenotype segregation in F2 progeny of the *abp1* knock-out x knock-down cross. Expected genotype and phenotype segregation ratios for three possible scenarios are shown. S/s = ethanol-inducible cassette positive/negative, A/a = wild-type *ABP1*/ *abp1* knock-out. Genotypes manifesting wild-type phenotype are shown on white background, KD phenotype on green background, genotypes that might exhibit both WT and KD phenotypes are on pale green background.

We crossed each of the conditional lines with *abp1*-TD1 and *abp1*-c1 null mutants and with an ABP1-WTc1 line as a control and analyzed seedling phenotypes of ethanol induced F2 segregating plants ( Figure 1a). We hypothesized that in case of an adaptive process, the conditional *abp1* KD phenotypes (short wavy roots and epinastic cotyledons) would not be manifested in homozygous *abp1* null background, resulting in a 9/16 KD and 7/16 WT phenotype segregation ratio. If the inducible phenotypes in the KD lines are independent of ABP1, these phenotypes will be manifested even in the absence of the functional *ABP1* gene, thus resulting in a classic Mendelian 3/4 KD and 1/4 WT phenotype segregation ratio. In case of the presence of background suppressive mutation(s), the KD phenotype segregation ratio would lie anywhere between 3/16 (dominant suppressor mutation closely linked to the *ABP1* locus) and 3/4 (recessive mutation with low penetrance and no linkage to *ABP1*) ( Supplementary Figure 1).

Segregation of the morphological phenotypes in the F2 plants from different crosses is summarized in Figure 1b. These observations show that strong phenotypes in both the *abp1* antisense-based and the scFv12-based conditional knock-down lines segregate approximately 75% in the F2 crosses with *abp1-c1*. This observation favors the scenario that the strong morphological defects in the KD lines are not influenced by the presence or absence of the functional *ABP1* gene copy. The F2 phenotypic segregation is however shifted in favor of WT-looking plants in all three KD lines crossed to *abp1-TD1*. This segregation shift may be ascribed to partial transcriptional silencing of the ethanol-inducible constructs due to the presence of multiple 35S promoters/enhancers in the constructs themselves as well as the tandem T-DNA insertion in *abp1-TD1*.

**Supplementary Figure 2.**
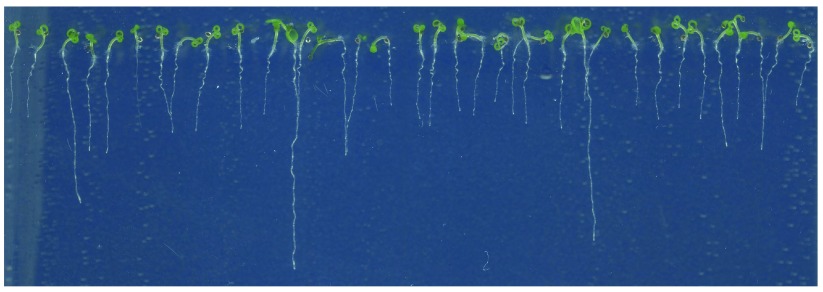
Restoration of the ethanol-inducible phenotype in the progeny of the F2 WT-looking plant from the cross *SS12K9 × abp1-c1* containing knock-down cassette and wild-type *ABP1* version. In the presence of 5% ethanol, F3 progeny show homogenous KD phenotype indicating that silencing of the construct might be responsible for wild-type phenotype of this plant in the F2 generation.

We genotyped all analyzed F2 plants for the presence of the *alcR* transcriptional regulator, which is an integral part of the ethanol-inducible system and verified that the observed morphological defects were indeed correlating with the presence of the *ABP1* KD constructs ( Figure 1c). About 5% of seedlings from all lines showed WT phenotype despite being positive for the presence of *alcR* or vice versa. As this phenomenon was independent of *ABP1* genetic background and could not be reproduced in F3 progeny ( Supplementary Figure 2), we put it down to biological variability and/or occasional silencing of the ethanol-inducible constructs.

### Strong morphological defects in conditional *abp1* knock-down alleles can be manifested in homozygous *abp1* knock-out alleles

To investigate whether the *abp1* KD phenotypes can be observed in the absence of a functional copy of the At4g02980 *ABP1* gene we further genotyped the respective *abp1* mutations in F2 seedlings of all crosses ( Figure 2). As summarized in Figure 2c, in all crosses we were able to identify multiple homozygous *abp1* mutants that showed the strong KD phenotype following ethanol induction. This analysis demonstrates that strong morphological phenotypes in *abp1* antisense-based ( *abp1-AS*) and scFv12 antibody-based ( *SS12S6, SS12K9*) conditional KD lines can be generated also in a null *abp1* background.

In case of the crosses *SS12K9 × abp1-c1* and *SS12K9 × abp1-TD1* we observed a lower level of allelic segregation between the *abp1* mutations and the KD construct in their F2 progeny ( Figure 2c). Out of 28 genotyped plants with WT phenotype, 24 (85.7%) were homozygous for *abp1* mutation and did not contain the ethanol-inducible KD cassette. These results point towards genetic linkage between these two loci, most likely caused by the positional effect of the KD cassette located close to the *ABP1* locus on the chromosome 4. Nevertheless, some level of genetic recombination was happening between the two loci in the crosses as demonstrated by the identification of three F2 *SS12K9 × abp1-c1* plants showing KD phenotype that were homozygous for *abp1-c1* mutation ( Figure 2c). This analysis confirms that also *SS12K9* conditional KD construct can generate strong morphological phenotypes in the homozygous *abp1* knock-out alleles despite the insertion position being linked to the *ABP1* locus. Altogether these data are consistent with results obtained by the other crosses and further support that morphological phenotypes in the *abp1* knock-down lines can be generated in the absence of the functional ABP1. 

### Analysis of F3 generation confirms *SS12K9*-induced strong morphological defects in absence of ABP1 function

Next we tested the occurrence of the strong KD-induced morphological phenotypes in the absence of the functional ABP1 in the next generation by analyzing the F3 progeny of two *SS12K9 × abp1-c1* plants showing strong KD phenotype. We confirmed that the F3 progeny was homozygous for the *abp1-c1* mutation and segregated the ethanol-inducible construct approximately in a 3:1 ratio ( Figure 3b). After induction with ethanol, the analyzed F3 population of the *SS12K9 × abp1-c1* plant A segregated into 27 plants (67.5%) with KD phenotype and 13 WT looking plants (32.5%) ( Figure 3). The F3 population of plant B segregated into 18 plants with KD phenotype (81.2%) and 4 WT looking plants (18.2%) (data not shown). Genotyping of all F3 plants with ethanol-inducible phenotypes revealed that they contain KD construct in the homozygous *abp1-c1* background ( Figure 3b). Notably, among the 17 analyzed WT looking F3 seedlings we also identified two plants that contain the ethanol-inducible construct in homozygous *abp1-c1* background ( Figure 3b) suggesting that in these plants the functionality of the construct was affected, most probably by its silencing. Nonetheless, the majority of the plants containing the ethanol-inducible construct generated the strong morphological phenotypes even in the *abp1 ^-/-^* homozygous background.

We also analyzed the *ABP1* expression in WT, *abp1-c1* and SS12K9 × *abp1-c1* F3 seedlings by quantitative RT-PCR just to verify that introducing KD alleles does not influence, in any way, the *ABP1* expression ( Figure 3c). We observed *ca.* 80% decrease in *ABP1* transcript levels in *abp1-c1.* We assume that this difference - somewhat surprising, since the *CRISPR*-induced small deletion does not necessarily decrease transcript levels - is probably caused by the decreased stability of the mutant *mRNA*. SS12K9 × *abp1-c1* F3 plants positive for the KD phenotype and homozygous for *abp1-c1* showed the same 80% decrease in *ABP1* transcription.

In summary, the phenotypic, genotypic and expression analyses consistently showed that all three conditional *abp1* knock-down alleles can generate strong morphological defects also in the absence of the functional ABP1 protein.

## Discussion

### Strong morphological phenotypes in *abp1* conditional knock-down alleles are not caused by ABP1 down-regulation

All three available conditional *abp1* knock-down alleles have been extensively characterized and used to link number of developmental and cellular processes to the ABP1-mediated signaling (for overview, see Grones & Friml, 2015). They are based on two unrelated strategies for down-regulation of the protein’s functionality: the antisense ( *abp1-AS*) and the scFv12 monoclonal antibody expression ( *SS12S6, SS12K9*), which suppress the protein functionality by entirely different mechanisms and at different levels ( Tromas *et al.*, 2009). All three lines showed consistent and reproducible results in a number of different laboratories and a number of developmental, physiological and cellular processes.

Nonetheless, our analysis, made possible by the newly available *abp1* knock-out lines ( Gao *et al.*, 2015), strongly suggests that these observed and described effects are not caused by conditional down-regulation of the ABP1. This is supported by the fact that all three constructs show the same strong conditional phenotypes in two different homozygous *abp1* null alleles. This means that even in the absence of the functional ABP1 protein, the ethanol-inducible constructs are inducing phenotypic defects that were originally ascribed to the down-regulation of ABP1. Therefore, results generated using these lines need to be critically re-interpreted.

### Possible modes of action of *abp1* conditional knock-down lines

All three types of *abp1* KD *Arabidopsis* lines generate indistinguishable morphological phenotypes. How it is possible that independent lines using fundamentally different approaches for functional down-regulation of a unique target would have in fact the same off-target effects; we do not know. One possible explanation is that the morphological defects are an artifact of the ethanol-inducible expression system. However, control lines generated in parallel using the same vector and expressing the *UIDA* reporter gene did not exhibit any significant growth and developmental alterations ( Braun *et al.*, 2008). Furthermore, a number of authors have used the same ethanol-inducible system to control the expression of distinct genes and to the best of our knowledge, there are no reports describing similar phenotypes by using the ethanol-inducible system for other genes in other studies ( Battaglia *et al.*, 2006; Deveaux *et al.*, 2003; Laufs *et al.*, 2003; Peaucelle *et al.*, 2008; Roslan *et al.*, 2001). This system was also used to successfully rescue mutant defects after ethanol induction of gene expression e.g. for LEAFY ( Maizel & Weigel, 2004) or for N-myristoyltransferase ( Pierre *et al.*, 2007) indicating that it is not responsible per se of the phenotypes observed with the ethanol inducible ABP1 AS and scFv12 constructs. In tobacco plants and BY-2 cells, tetracycline de-repressible promoter-driven expression of the *SS12S* and *SS12K* constructs resulted in similar growth defects as their ethanol-inducible expression in *Arabidopsis* ( Braun *et al.* 2008; David *et al.*, 2007), suggesting that the observed phenotypes are tightly correlated to the scFv12 action *.* The expression of the scFv12 in the cytosol had however no effect on cell proliferation in BY2 cells indicating that expression of scFv12 *per se* is not sufficient to generate severe phenotypes whatever its cellular localisation and that scFv12 effects are correlated to its secretion and/or retention in the ER that are known location of ABP1 ( David *et al.*, 2007).

Another possibility is that both the antisense- and antibody-based lines have off-target(s) either on the very same gene(s) or elements of a common genetic pathway. Such a hypothesis would be supported by strict similarities in the phenotypes resulting from ABP1 antisense and scFv12 expression and by the fact that opposite and auxin-related defects were observed in both constitutive and conditional gain-of-function *Arabidopsis* transgenic plants as well as transitionally expressing tobacco cells ( Grones *et al.*, 2015; Robert *et al.*, 2010). ABP1 is placed within the superfamily of cupins based on the presence of cupin-like motifs HXH(X) _11_G and P(X) _4_H(X) _3_N (where X is any amino-acid residue) and a β-barrel jellyroll fold subunit structure ( Dunwell *et al.*, 2004; Woo *et al.*, 2002). The epitope recognized by the scFv12 might be present in proteins belonging to this functionally highly diverse protein superfamily. On the other hand, the sequence similarity of even the closest *ABP1* homologues in *Arabidopsis* does not seem to be sufficiently high to be targeted by the *abp1-AS* constructs, thus this explanation is unlikely as well.

We also cannot completely rule out that the WT phenotype of the *abp1* knock-out mutants is caused by suppressor mutation(s). However, we do not consider it very likely, as this would imply that the similar mutation(s) or mutations with similar effects are present in the genetic background of both *abp1-c1* and *abp1-TD1,* which are independent alleles from independent mutant collections.

In summary, we do not understand how it is possible that the used *abp1* knock-down alleles generate the similar strong morphological phenotypes also in absence of the functional ABP1 protein. All possible explanations we could come up with are unlikely, including common off-targets in *abp1* antisense and antibody KD lines or common suppressor mutations in two different *abp1* knock-out alleles. Thus, more experimentation is needed to figure out what really happens in the different *abp1* KD lines and how it is possible that they independently generate phenotypes that are so consistent. Whatever the explanation at the end will be, in light of the presented data it seems obvious that these lines do not act solely by down-regulating the ABP1 function, despite the accumulation of well-fitting data from independent and complementary approaches. It is a sobering realization that even when you use independent approaches with all standard controls performed, there is no real guarantee that the observations will not lead you amiss.

## Data availability

The data referenced by this article are under copyright with the following copyright statement: Copyright: © 2016 Michalko J et al.

Data associated with the article are available under the terms of the Creative Commons Zero "No rights reserved" data waiver (CC0 1.0 Public domain dedication). 

*F1000Research*: Dataset 1. Scans of ethanol-induced F2 seedlings of crosses (A) *SS12S6 × abp1-c1*, (B) *SS12S6 × abp1-TD1*, (C) *abp1-AS × abp1-c1*, (D) *abp1-AS × abp1-TD1*, (E) *SS12K9 × abp1-c1* and (F) *SS12K9 × abp1-TD1* that were used for phenotyping and genotyping ( Figure 1 and Figure 2)., 10.5256/f1000research.7654.d110722 ( Michalko *et al.*, 2016a).

*F1000Research*: Dataset 2. Agarose gel images from the PCR genotyping of the F2 crosses (A) *SS12S6 × abp1-c1*, (B) *SS12S6 × abp1-TD1*, (C) *abp1-AS × abp1-c1*, (D) *abp1-AS × abp1-TD1*, (E) *SS12K9 × abp1-c1* and (F) *SS12K9 × abp1-TD1* ( Figure 3), 10.5256/f1000research.7654.d110723 ( Michalko *et al.*, 2016b).

*F1000Research*: Dataset 3. Source qPCR data ( Figure 3c), 10.5256/f1000research.7654.d110724 ( Michalko *et al.*, 2016c).
