# Supplementary material for: Strong morphological defects in conditional Arabidopsis abp1 knock-down mutants generated in absence of functional ABP1 protein
Source: F1000Res. 2016 Jan 20;5:86. [Version 1] doi: 10.12688/f1000research.7654.1 (PMC4748827; doi:10.12688/f1000research.7654.1)
Supplement: Agarose gel images from the PCR genotyping of the F2 crosses (A) SS12S6 × abp1-c1, (B) SS12S6 × abp1-TD1, (C) abp1-AS × abp1-c1, (D) abp1-AS × abp1-TD1, (E) SS12K9 × abp1-c1 and (F) SS12K9 ×abp1-TD1 (Figure 3) — All crosses were genotyped for the presence of the alcR transcriptional regulator (first row of the gel images) which is an integral part of the ethanol-inducible cassette in abp1 knock-down lines. The presence of point mutation in abp1-c1 crosses was genotyped by restriction analysis of ABP1 PCR product as described in Gao et al. (2015) (second row of the gel images of abp1-c1 crosses). The presence of the T-DNA insertion in abp1-TD1 crosses was genotyped according to Gao et al. (2015) (second and third row of gel images of abp1-TD1 crosses). GeneRuler DNA ladder mix #0331 (Thermo Scientific) was used as a fragment size standard to determine the approximate size of DNA fragments. Fragment sizes of 1000 bp and 500 bp are indicated. [file f1000research-5-8243-s0001.tgz › 46464419-951f-452d-bb81-327ee613a7b4_Data_2.zip!/Data 2/Dataset_2_E.pdf]

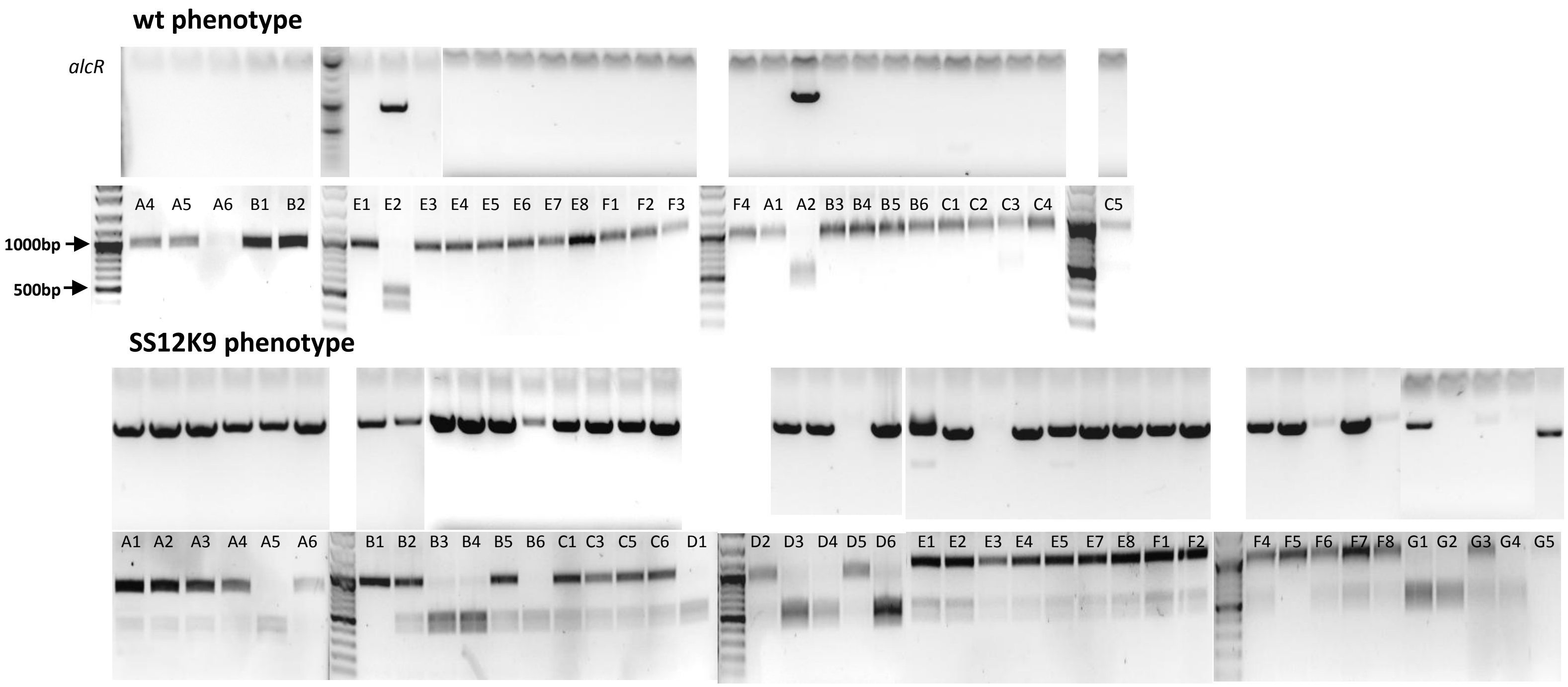
wt phenotype
alcR
A4 A5 A6 B1 B2 E1 E2 E3 E4 E5 E6 E7 E8 F1 F2 F3 F4 A1 A2 B3 B4 B5 B6 C1 C2 C3 C4 C5
1000bp
500bp
SS12K9 phenotype
A1 A2 A3 A4 A5 A6 B1 B2 B3 B4 B5 B6 C1 C3 C5 C6 D1 D2 D3 D4 D5 D6 E1 E2 E3 E4 E5 E7 E8 F1 F2 F4 F5 F6 F7 F8 G1 G2 G3 G4 G5
