# Supplementary material for: Strong morphological defects in conditional Arabidopsis abp1 knock-down mutants generated in absence of functional ABP1 protein
Source: F1000Res. 2016 Jan 20;5:86. [Version 1] doi: 10.12688/f1000research.7654.1 (PMC4748827; doi:10.12688/f1000research.7654.1)
Supplement: Peer review history

# Peer review: Referee response for version 1

**DOI:** 10.5256/f1000research.8243.r12003

**Published:** 2016-02-09

**Authors:** Christian Luschnig

**Affiliations:** 1 Department of Applied Genetics and Cell Biology, University of Natural Resources and Life Sciences, Vienna, Austria

In their m/s, the authors present an analysis of genetic interactions between published *ABP1* knockdown lines (based on antisense and immune-suppression approaches) and the *Arabidopsis* *ABP1* locus. After crossing three different knockdown lines into recently described *abp1* knockout alleles, followed by segregation analysis of resulting F2 and F3 progeny, the authors came to the conclusion that growth defects of their knockdown lines do not depend on a functional *ABP1* locus.

These findings represent a valuable contribution to ongoing attempts, trying to clarify *ABP1* issues. Nevertheless, off-target loci recognized in the knockdown lines remain mysterious. Are there any ABP1-related loci (cupins?), expression of which could be affected by these knockdown lines? Perhaps this could be tested in the *abp1-AS* line.

Phenotypes of the knockdown lines are reminiscent of mutants with altered auxin responses. Did the authors look into expression of some of the characterized auxin-related loci? This could give us a better idea about the genetic determinants, causing the phenotypes in these knockdown lines.

I have read this submission. I believe that I have an appropriate level of expertise to confirm that it is of an acceptable scientific standard, however I have significant reservations, as outlined above.

# Peer review: Referee response for version 1

**DOI:** 10.5256/f1000research.8243.r12004

**Published:** 2016-02-05

**Authors:** Richard M. Napier

**Affiliations:** 1 School of Life Sciences, University of Warwick, Coventry, UK

The authors have produced a very thorough analysis of a set of well-known conditional ABP1 knock-down Arabidopsis lines. Using crosses with the recently-described abp1 knock-out lines from the work of Gao *et al. *(2015), the manuscript reveals that the phenotypes previously associated with loss of ABP1 can be induced even in genetic backgrounds that lack ABP1. These findings cover results from the two independent strategies used to create the conditional knock-down phenotypes, conditional immunosuppression and conditional antisense expression.

The assay used for this work was Arabidopsis root growth, not the very detailed phenotypes described recently from the conditional lines such as PIN endocytosis, ROP GTPase activation or microtubule rearrangement. However, impaired root growth was part of the initial suite of phenotypes associated with induced knock-down. The assays show very clearly that growth impairment correlates with the inheritance of the inducible cassette even in the absence of a functional ABP1 gene. We can surmise that the detailed subcellular phenotypes listed above are associated with the gross morphological changes recorded here.  There is clearly some intriguing physiology associated with the switch induced by ethanol treatment, but ABP1 is not part of that story. 

The title and abstract are appropriate (but see note below*), the work is done well, the data is presented clearly and fully, the text is very well structured and is easy to follow. There is a thorough introduction which explains how and why experimentation developed to tackle tangible problems linked to existing knowledge and understanding of ABP1 genetic resources.  It is shown that the science was not misled by carelessness or device, that a spectrum of consistencies coloured the hypotheses covering ABP1 activity, and that these hypotheses have now been proven incorrect thanks to improved technologies, vigilance and critical reappraisal.

*The abstract ends with a sentence “…asks for further reflections on the biological function of ABP1 or alternative explanations for the missing phenotypic defects in the abp1 loss-of-function alleles.”  I think that this would be better if it read “… AND alternative explanations for the intriguing phenotypes previously associated with loss of ABP1 activity”. Or similar. I understand that there is a requirement on all sides of the debate to be objective and critical about their data and no harm is done by registering this. However, the balance of probabilities needs to be recognised and, in my view, much more has to be gained from encouraging discovery of the root cause of the many fascinating phenotypes thrown up by the ABP1 KD lines than by searching absent defects. Therefore a small rewording at the end of the abstract is recommended.  Otherwise I have no changes to suggest.

I have read this submission. I believe that I have an appropriate level of expertise to confirm that it is of an acceptable scientific standard.

# Peer review: Referee response for version 1

**DOI:** 10.5256/f1000research.8243.r12006

**Published:** 2016-01-26

**Authors:** Lars Ostergaard

**Affiliations:** 1 Department of Crop Genetics, John Innes Centre, Norwich, UK

In this manuscript, Michalko *et al.* investigates the basis of the developmental phenotypes observed in knock-down (KD) lines of the *Arabidopsis ABP1* gene. By analysing segregation of defects in root elongation in the F2 and F3 generation of crosses between three KD *abp1* alleles and two recently reported *abp1* knock-out (KO) alleles, they conclude that the previously reported phenotypes of the KD lines are not due to loss of ABP1 function. The manuscript is clearly written and the experiments are thoroughly carried out providing an important contribution to the ABP1 saga.

Although I sympathise with the authors difficulty in identifying an explanation for how the KD lines can lead to the observed abnormalities, one experiment that should be done would in my opinion be a test of the expression of the closest *ABP1*-like genes that can be identified.

I have read this submission. I believe that I have an appropriate level of expertise to confirm that it is of an acceptable scientific standard, however I have significant reservations, as outlined above.
